# Unveiling SSR4: a promising biomarker in esophageal squamous cell carcinoma

**DOI:** 10.3389/fimmu.2025.1544154

**Published:** 2025-02-24

**Authors:** Jiaqi Zhang, Fang Jia, Chuqiao Li, Shunzhe Song, Aixia Gong

**Affiliations:** ^1^ Department of Digestive Endoscopy, The First Affiliated Hospital of Dalian Medical University, Dalian, Liaoning, China; ^2^ Department of Gastroenterology, The First Affiliated Hospital of Jinzhou Medical University, Jinzhou, Liaoning, China

**Keywords:** esophageal squamous cell carcinoma, SSR4, biomarker, single-cell transcriptomics, immune cells

## Abstract

**Background:**

Esophageal squamous cell carcinoma (ESCC) represents a frequent cancer with a poor prognosis. Altered glucose metabolism contributes factor to ESCC progression. In our previous study, signal sequence receptor subunit delta (SSR4) was included in an ESCC prognostic model; however, the mechanisms underlying SSR4 implication in ESCC remain ambiguous. Accordingly, we aim to determine the interconnection between SSR4 expression and clinical characteristics of ESCC.

**Methods:**

This differential expression and prognostic significance of SSR4 was performed using bulk RNA-seq data and 110 patients with complete follow-up information. The ESCC cell subsets with the highest gene expression levels were identified with single-cell data. Gene function and enrichment, immune infiltration, cell communication, and molecular docking analyses were performed.

**Results:**

Unlike adjacent non-cancerous tissues, SSR4 was overexpressed in ESCC tissues, validated by both reverse transcription-qPCR and IHC staining. SSR4 expression was related to the N stage, lymph node metastasis, and AJCC TNM classification stage. Patients exhibiting low SSR4 expression had a more favorable prognosis. The highest SSR4 expression was recognized in tumor plasma cells. Continued exploration of immune infiltration highlighted a close association between SSR4 gene expression and the infiltration of immune cells such as plasma cells. On dividing cells into SSR4-positive and -negative groups, CellChat analysis indicated that SSR4 may regulate the interactions that existed between ESCC tumor plasma cells and the tumor microenvironment (TME) by modulating the MIF/CD74/CXCR4 axis.

**Conclusion:**

The SSR4 gene may have significant relevance with clinical pathological factors, and play a critical role in the regulation of tumor microenvironment of ESCC patients. Overall, SSR4 may be a promising ESCC biomarker with prospective applicability in clinical diagnosis as well as the development of targeted treatment approaches in patients of ESCC.

## Introduction

Among all aggressive malignancies, esophageal cancer (ESCA) remains to be a major driver of mortality (1). Most patients with ESCA suffer from esophageal adenocarcinoma or esophageal squamous cell carcinoma (ESCC), which is frequent in Asia and East Africa (2). Treatment for advanced-stage ESCC is rigorous and may include chemotherapy, chemoradiotherapy, surgery, or combinations of these therapies (3). Endoscopic screening constitutes the gold standard for ESCC identification, and the application of Lugol’s iodine solution can assist in detecting ESCC. During this procedure, the glycogen-containing mucosal cells stain brown, whereas the glycogen-depleted neoplastic cells stain pink. Therefore, it has been proposed that defective glycogen synthesis and excessive glycogenolysis contribute to ESCC development and progression instead of being merely due to cellular neoplastic transformation (4). Nonetheless, the underlying mechanism remains vague. Due to lacking approved targeted therapies and the absence of clinically significant biomarkers for the early diagnosis of ESCC (5), identifying more ESCC prognostic biomarkers and therapeutic targets is necessary.

In our previous study, signal sequence receptor subunit delta (SSR4) was involved in a prognostic ESCC model (6), which was constructed with the glucose metabolism gene set because the cellular glycogen is lost in the early stages (mild squamous dysplasia or low-grade intraepithelial neoplasia) and continues until invasive ESCC. The SSR4 encodes the delta subunit of the translocon-associated protein (TRAPδ) complex that contributes to protein translocation through the endoplasmic reticulum (ER) membrane. The TRAP complex reportedly participates in governing humoral immunity through immunoglobin secretion and transport (7), which plays a crucial role in the N-glycosylation pathway (8). Diseases associated with SSR4 mutations include two types of congenital glycosylation disorders (8). The SSR4 is overexpressed in immune cells of the tumor microenvironment (TME) in colon adenocarcinoma and gastric cancer (9, 10) and may serve as a prognostic biomarker that can predict better overall survival (OS) and therapeutic efficiency in patients with colon adenocarcinoma. These findings suggested that SSR4 is crucial in ESCC progression. Nevertheless, SSR4 expression in ESCC, its relation with clinical factors, and its potential role in ESCC have not been fully elucidated.

Contemporary biomedical research relies heavily on the application of bioinformatics for clinical diagnosis, treatment decision-making, and finding new targets. Research on single-cell transcriptomics in ESCC has rapidly developed, revealing the intricate cellular dynamics associated with malignancies (11).

In this study, a comprehensive analysis and validation of differentially expressed SSR4 and its prognostic significance were conducted using public databases and immunohistochemistry (IHC). Our aim is to explore the interconnection between SSR4 expression and the clinical attributes of patients who have ESCC to determine the potential of SSR4 as a biomarker of prognosis. Additionally, using scRNA-seq data from ESCC, we identified the ESCC cell subsets that exhibited the highest gene expression levels. This was followed by gene function and enrichment, cell communication, and molecular docking analyses. This underscores the pivotal role of SSR4 in ESCC and implies that SSR4 has significant potential as a biomarker for ESCC. Consequently, using this technology in clinical diagnosis and developing targeted therapeutics is recommended.

## Materials and methods

### Data preparation and processing

The TCGA datasets of ESCC were obtained by accessing the TCGA data portal (https://tcga-data.nci.nih.gov/tcga/). The NCBI is liable for developing and maintaining the GEO database (https://www.ncbi.nlm.nih.gov/geo/info/datasets.html). Data files for GSE188900 were sourced from the NCBI GEO public repository and encompassed the single-cell expression profiles of five patients who have ESCC (12). This dataset included one normal case and seven ESCC samples utilized in the analysis. The GTEx data were obtained from the V8 version; detailed information can be accessed on the official GTEx website (https://gtexportal.org/home/datasets).

### Expression and prognostic analysis of SSR4

RNA-seq expression profiles were converted from FPKM format to TPM format, and log2 was transformed for analysis. Data were obtained only from patients with clinical survival information. After retaining samples that included both RNA-seq data and clinical data, 82 samples were selected for further analysis. Utilizing the log-rank test, we evaluated differences in survival outcomes between both groups within the Kaplan–Meier (K-M) survival analysis. Additionally, survival analysis was carried out via the survival and survminer packages in the R software (R Foundation for Statistical Computing, Vienna, Austria).

### Sample collection

Tissue microarray slides were procured from Shanghai Outdo Biotech Co. Ltd. (Shanghai, China; No. HEsoS180Su09). The final statistical analysis included 110 ESCC samples and 68 benign lesions from 112 ESCC samples after excluding those with incomplete clinical data. Prior to surgery, no patients received radiotherapy, chemotherapy, or other antitumor treatments. Histological classification of tumor stages was done using the AJCC TNM staging system (13). All patients were followed up for 1.75–3.4 years, and the follow-up was terminated in July 2015. All study patients signed informed consent, and the local ethics committee authorized the study (No. SHYJS-CP-1907002). This study was exempted from ethical review by the Ethics Committee of the First Affiliated Hospital of Dalian Medical University.

### Cell culture

Human ESCC cell lines, KYSE-270 and KYSE-70, were procured from Qingqi Bio (Shanghai, China) and cultured in Dulbecco’s Modified Eagle Medium (DMEM) with high glucose. The TE-1 human ESCC cell line was acquired from Punuosai Co., Ltd. (Wuhan, China) and maintained in RPMI-1640 medium. The normal human esophageal squamous epithelial cell line, Het-1A, was sourced from Beina Bio (Beijing, China) and cultured in Bronchial Epithelial Cell Basal Medium (BEBM). All cell lines were incubated at 37°C in a humidified atmosphere containing 5% CO2.

### Reverse transcription-qPCR

To assess the transcriptional levels of SSR4 mRNA, RNA was extracted from esophageal cell lines with the use of an RNA extraction buffer (G3013, Servicebio, China). This was followed by reverse transcription employing the Servicebio RT First Strand cDNA Synthesis Kit (G3330, Servicebio).

cDNA was amplified using SYBR green qPCR master mix (Servicebio, G3320) buffer system via fluorescence quantitative PCR instrument (ABI 7500, USA). The expression data were normalized to the housekeeping gene β-actin using the ΔCt method. Relative expression levels of the target genes were calculated using the 2−ΔΔCt method, with the expression level in the control group set as 1.0. The primer sequences utilized in this study are detailed in **Supplementary Table S1**.

### IHC staining

The primary antibody used was an anti-SSR4 antibody from Proteintech (San Diego, CA, USA) (11655-2-AP). After 8 min of ceasefire, the antigen was repaired with medium fire for 8 min to boiling point and then on medium-low heat for 7 min. H-scores (range 0–300) were used to measure the IHC staining intensity. In the event of discrepancies in the results, a third authoritative pathologist was consulted to interpret the findings, engage in discussions, or calculate the average value, thereby arriving at a final conclusion.

### Quality control and cell annotation of sc-RNA seq data of ESCC

Initially, the R package “Seurat” was deployed to identify and exclude samples with aberrant expression profiles. In this study, the data samples were initially filtered based on gene counts (nFeature_RNA), sequencing depth (nCount_RNA), and mitochondrial gene percentage (percentage) to ensure data reliability. The filtering criteria were as follows: nFeature_RNA > 200 and < 7500 percent.mt < 15, and nCount_RNA < 50,000. To minimize the impact of sequence depth, technical variation, and efficient capture of data, the data were standardized and normalized. PCA was performed to reduce noise, and a scree plot was employed to define the optimal number of principal components. Using a uniform manifold approximation and projection (UMAP) for nonlinear dimensionality reduction (14), the spatial configuration of each cluster was ascertained. The FindNeighbor and FindCluster functions in the R package “Seurat,” and the R package “SingleR” combined with the CellMarker database (15) were used to export the cell type annotation results.

### Differential expression genes screening

Genes with differential expression were screened through the R packages “limma” and “DESeq2.” The DEG analysis was performed between the SSR4-positive (SSR4+) and SSR4-negative (SSR4-) groups. Genes were classified as differentially expressed if they satisfied an absolute log2 fold change > 1.0 and a false discovery rate < 0.05.

### Analysis of SSR4 immune infiltration

The extent of immune cell infiltration within the samples was quantified utilizing single-sample Gene Set Enrichment Analysis (ssGSEA) (16). Subsequently, to examine the interrelationships among immune cells the SSR4 gene, the Spearman correlation coefficient was determined employing the R packages “corrplot”.

### SSR4-DEGs enrichment analysis

Functional enrichment analyses were conducted using GO and KEGG (17). The “AUCell” package was deployed to score the regulon activity in each cell.

### Cell-cell communication analysis

The R package, “CellChat,” was employed to determine probable interactions between SSR4+ and SSR4- cells in ESCC tumor plasma cell populations and other cells in the TME of ESCC. This package simulates intercellular communication considering interactions between ligands, receptors, and their cofactors (18).

### Construction of a 3D structure model of SSR4 protein and virtual screening of potential drugs

The 3D structure of SSR4 was derived from the AlphaFold Protein Structure Database (https://alphafold.com/) (19). The Comparative Toxicogenomics Database (https://www.ctdbase.org/) offers data on chemical–gene/protein interactions, chemical–disease relationships, and gene–disease relations (20). The ingredient structures were sourced by accessing the PubChem database (http://pubchem.ncbi.nlm.nih.gov/). Molecular docking was conducted through the Lamarckian genetic algorithm implemented in AutoDock 4.2 software (https://autodock.scripps.edu/downloads/) 50 times, choosing the result with the lowest binding energy for further analysis. PyMOL visualization (https://www.pymol.org/) was created based on the docking results (21).

### Statistical analysis

Data analysis was carried out through IBM SPSS Statistics (version 24.0; SPSS Inc., IBM Corp., Armonk, NY, USA), GraphPad Prism (version 5; GraphPad Software; https://www.graphpad.com/), and R software (version 4.3.2). Continuous variables are presented as mean, median, standard deviations, and interquartile range while reporting categorical variables as frequency or percentage. The data normality was initially assessed utilizing the D’Agostino and Pearson omnibus test or the Shapiro–Wilk test. For intragroup comparisons of continuous variables, either the paired t-test or Wilcoxon signed-rank test was applied, contingent upon the data distribution. Conversely, intergroup comparisons were performed with either the independent sample *t*-test or the Mann–Whitney U test. The chi-square test was deployed for categorical variables. Survival curves were analyzed by the K-M method. The effect of clinicopathological features and gene expression on OS was assessed employing the Cox proportional hazards model. A two-tailed *P* < 0.05 was deemed statistical significance.

## Results

**Figure 1:**
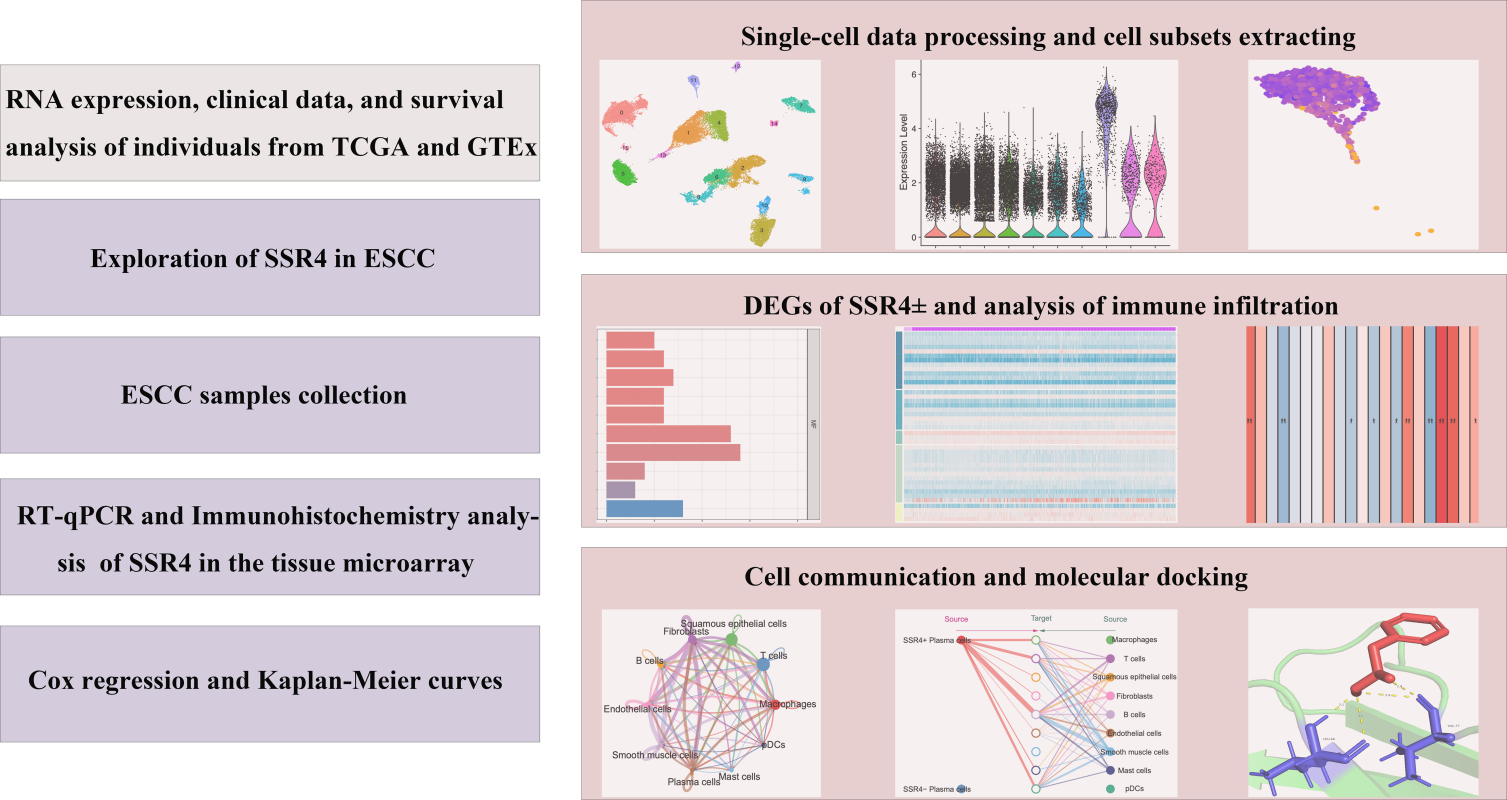
Study flowchart. ESCC, esophageal squamous cell carcinoma; SSR4, signal sequence receptor subunit delta; DEGs, differentially expressed genes.

The research flowchart of this research was shown in **Figure 1**.

### Highly expression of SSR4 in ESCC tissues

**Figure 2:**
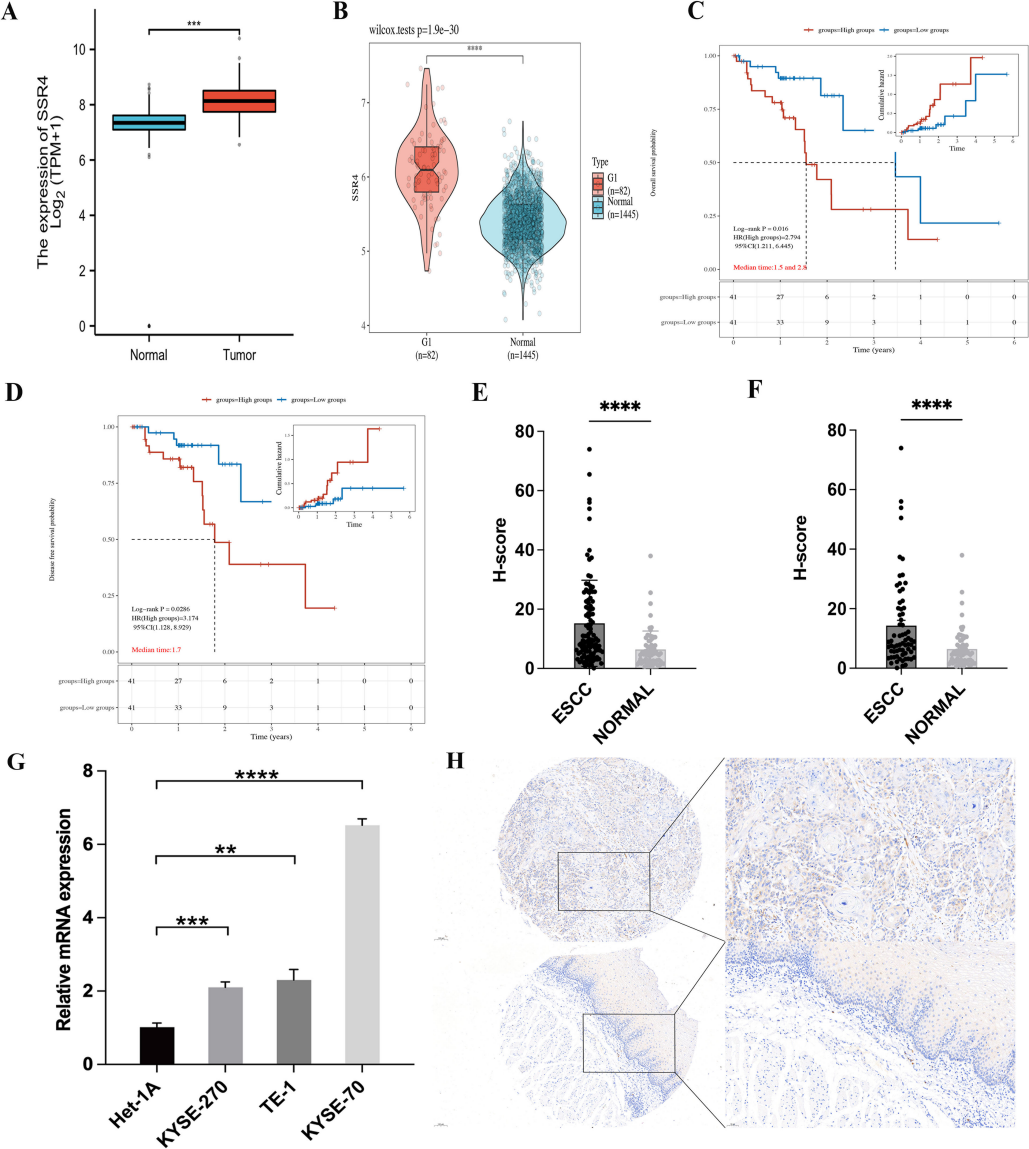
Signal sequence receptor subunit delta (SSR4) identification in esophageal squamous cell carcinoma (ESCC) patients. **(A)** The Cancer Genome Atlas (TCGA) database: Difference expression of SSR4 in ESCA and para-cancerous (*P* < 0.001). **(B)** TCGA and Genotype-Tissue Expression (GTEx) databases: Difference in expression of SSR4 in ESCC and healthy individuals (*P* < 0.0001). **(C)** High SSR4 expression was related to poor overall survival (*P* = 0.016) and **(D)** poor disease-free survival in ESCC patients (*P* = 0.0286). **(E)** SSR4 numbers in ESCC tissues and adjacent tissues (*P* < 0.0001) and **(F)** matched para-cancerous tissues (*P* < 0.0001). **(G)** Results of RT-qPCR demonstrated that SSR4 was highly expressed in ESCC cell lines. Analysis was performed on data from three independent experiments. **(H)** Tissue microarray and immunohistochemistry staining of SSR4 in ESCC and adjacent tissue (magnification x10 and x20; scale bar: 100 and 50 µm, respectively). *Notes*: DEGs, differentially expressed genes; ESCA, esophageal carcinoma. (**P < 0.01, ***P < 0.001, ****P < 0.0001.).

Based on TCGA and GTEx databases, differential expression of SSR4 in ESCA tissues (n = 182) and normal esophageal squamous epithelia (n = 666) was observed (*P* < 0.001, **Figure 2A**). Furthermore, significantly higher differential expression of SSR4 was observed in ESCC tissues (n = 82), unlike normal tissues (n = 1445, *P* < 0.0001, **Figure 2B**).

### Highly expression of SSR4 in ESCC tissues related to poor prognosis

Survival analysis showcased that SSR4 overexpression was significantly associated with OS and shorter disease-free survival (DFS; *P* = 0.016 and *P* = 0.0286, respectively), indicating that patients who have ESCC with overexpressed SSR4 have a poor prognosis (**Figures 2C, D**).

### Validation of RT-qPCR and microarray stained by immunohistochemistry

An IHC study was conducted on 112 ESCC tissues and 68 para-cancer tissues from the tissue microarray, demonstrating significantly increased SSR4 expression in ESCC tissues (*P* < 0.0001) compared to normal tissues (**Figure 2E**). In addition, **Figure 2F** demonstrated that in 68 paired ESCC and adjacent tissues, SSR4 was overexpressed in cancer tissues more than in the adjacent tissues (*P* < 0.0001, **Figure 2F**). The RT-qPCR results demonstrated that SSR4 expression was significantly elevated in ESCC cell lines (KYSE-270, TE-1, KYSE-70) compared to normal esophageal squamous epithelial cells (Het-1A), with statistical significance observed at *P*<0.001, *P*<0.01, and *P*<0.0001, respectively (**Figure 2G**). **Figure 2H** depicts the IHC results. We further analyzed the expression of PD-L1 and PD-1, a key immune checkpoint molecule, in tumor versus normal tissues. As shown in **Supplementary Figure S1**, PD-L1 expression was significantly higher in ESCC tissues compared to normal tissues (*P* < 0.001), consistent with its role in tumor immune evasion.

### SSR4 is closely related to clinical and pathological findings and ESCC patients’ poor prognosis

An ESCC tissue microarray (n=112) was used to detect the correlation between SSR4 gene expression and clinical and pathological findings and ESCC patients’ prognosis. After excluding two patients with incomplete data, we analyzed 110 patients with complete data. Baseline information including age at diagnosis, sex, surgery time, survival status, follow-up data, OS, TNM tumor stage, AJCC TNM stage, and lymph node metastasis (LNM) was summarized in **Table 1**. Among these, 61 patients had high SSR4 expression, and 49 patients had low SSR4 expression. No evident associations were recognized between age, sex, tumor morphology, T stage, M stage, and SSR4 expression (*P* > 0.05), but were related to N stage (*P* < 0.001), LNM (*P* < 0.001), and AJCC TNM stage (*P* < 0.001, **Table 2**). Employing univariate and multivariate Cox regression analyses, we examined whether SSR4 expression was a prognostic factor. The results of univariate regression analysis elucidated that sex (*P* = 0.234), patient age at diagnosis (*P* = 0.002), morphology (*P* = 0.659), LNM (*P* = 0.004), LNM ratio (*P* < 0.001), T stage (*P* = 0.770), N stage (*P* = 0.004), M stage (*P* = 0.215), AJCC TNM stage (*P* = 0.006), and SSR4 expression (*P* = 0.022) were associated with OS (**Table 3**). Multifactor models were constructed using all meaningful univariate indices (*P* > 0.05). The multivariate analysis suggested that age (*P* = 0.001) and LNM ratio (*P* = 0.025) were independently as well as significantly associated with OS (**Table 3**). The K-M and log-rank tests indicated that the groups with high SSR4 expression survived for an average of 30 months, whereas those with low SSR4 expression survived for an average of 27 months. Unlike the low expression group, the high SSR4 expression group displayed significantly reduced OS (*P* = 0.03, **Supplementary Figure S2**).

**Table 1:** Baseline information of included individuals.

Clinical factors	N
Age	
<61	87 (79.09%)
≥ 61	23 (20.91%)
Gender	
Male	93 (84.55%)
Female	17 (15.45%)
Morphology	
Medullary	47 (42.73%)
Narrow	2 (1.82%)
Mushroom Umbrella	9 (8.18%)
Ulcer	52 (47.27%)
Lymph node metastasis	
No	61 (55.45%)
Yes	49 (44.55%)
T stage	
T1	2 (1.82%)
T2	25 (22.73%)
T3	79 (71.82%)
T4	4 (3.64%)
N stage	
N0-N1	90 (81.82%)
N2-N3	20 (18.18%)
M stage	
M0	109 (99.09%)
M1	1 (0.91%)
AJCCTNM	
1-2	66 (60.00%)
3-4	44 (40.00%)

**Table 2:** Relationship between signal sequence receptor subunit delta (SSR4) expression in esophageal squamous cell carcinoma (ESCC) and clinicopathological parameters.

Clinical factors	Gene expression		Statistics	*P*-value
	Low (N=61)	High (N=47)		
**Age**			0.010	0.904
<61	49 (80.33%)	38 (77.55%)		
≥ 61	12 (19.67%)	11 (22.45%)		
**Gender**			1.050	0.306
Male	54 (88.52%)	39 (79.59%)		
Female	7 (11.48%)	10 (20.41%)		
**Morphology**			**-**	0.633
Medullary	23 (37.70%)	24 (48.98%)		
Narrow	1 (1.64%)	1 (2.04%)		
Mushroom Umbrella	5 (8.20%)	4 (8.16%)		
	
Ulcer	32 (52.46%)	20 (40.82%)		
**Lymph node** **Metastasis**			32.07	***<0.001***
			
No	49 (80.33%)	12 (24.49%)		
Yes	12 (19.67%)	37 (75.51%)		
**T stage**			–	0.666
T1	1 (1.64%)	1 (2.04%)		
T2	15 (24.59%)	10 (20.41%)		
T3	44 (72.13%)	35 (71.43%)		
T4	1 (1.64%)	3 (6.12%)		
**N stage**			**-**	***<0.001***
N0-N1	59 (96.72%)	31 (63.27%)		
N2-N3	2 (3.28%)	18 (36.73%)		
**M stage**			**-**	0.445
M0	61 (100.00%)	48 (97.96%)		
M1	0 (0.00%)	1 (2.04%)		
**AJCCTNM**			21.71	***<0.001***
1-2	49 (80.33%)	17 (34.69%)		
3-4	12 (19.67%)	32 (65.31%)		

Bold and italic fonts indicate statistically significant between the two groups.

**Table 3:** Univariate and multivariate Cox analysis: correlation between signal sequence receptor subunit delta (SSR4) expression and the clinical characteristics of esophageal squamous cell carcinoma (ESCC) patients.

Clinical factors	Univariate cox		Multivariate cox	
	HR (95%CI)	*P*	HR (95%CI)	*P*
Gender	1.661 (0.720,3.831)	0.234	–	–
Age	3.059 (1.517,6.172)	*0.002*	3.275 (1.588,6.753)	*0.001*
Morphology	0.984 (0.747,1.203)	0.659	–	–
Lymph node metastatic	2.882 (1.395,5.951)	*0.004*	1.507 (0.295,7.703)	0.623
Lymph node metastatic ratio	34.132 (7.241,160.901)	*<0.001*	21.582 (1.466,317.765)	*0.025*
T stage	0.908 (0.476,1.732)	0.770	–	–
N stage	2.876 (1.391,5.943)	*0.004*	0.973 (0.297,3.188)	0.964
M stage	3.537 (0.481,26.038)	0.215	–	–
AJCCTNM	2.640 (1.312,5.312)	*0.006*	0.864 (0.179,4.160)	0.855
SSR4	2.271 (1.128,4.573)	*0.022*	1.507 (0.637,3.566)	0.351

Italic fonts indicate statistically significant.

### ScRNA-seq data analysis and single-cell transcriptome atlas of ESCC

**Figure 3:**
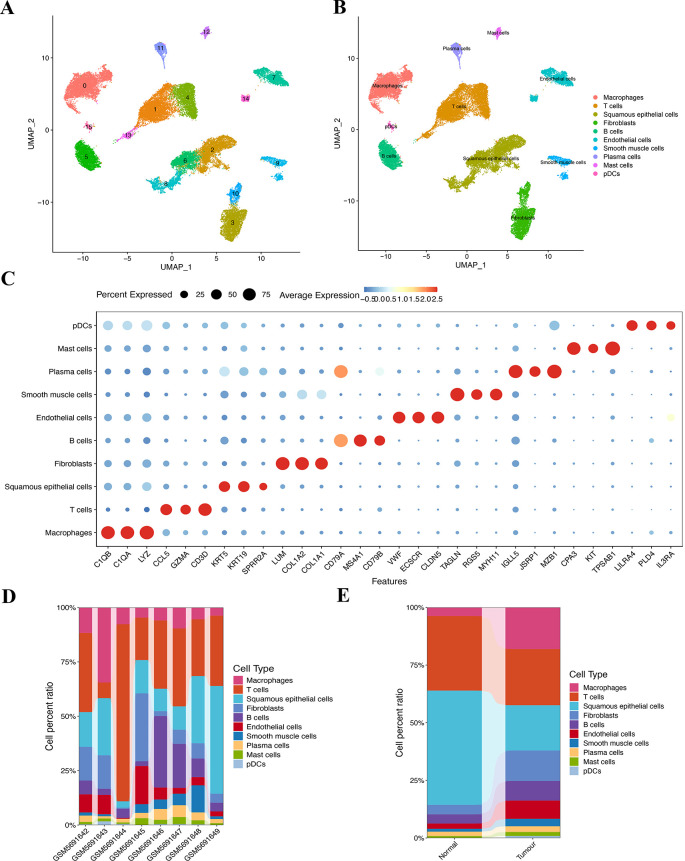
Clustering and annotation of the ESCC cells. **(A, B)** Sixteen clusters were annotated into 10 cell types. **(C)** The bubble chart shows specific markers corresponding to 10 different cell types. **(D)** Variations in the content percentages of 10 cell types in 8 groups of samples. **(E)** Cell distributions differ between individual samples in control and disease groups.

Here, 56,421 single cells were obtained after the scRNA-seq data underwent standardization, homogenization, PCA, and harmony analysis (**Supplementary Figures S3A–D**). UMAP analysis identified 16 cell clusters among the 37,020 single cells (**Figure 3A**). Each subtype was further annotated as macrophages, T cells, squamous epithelium, fibroblasts, B cells, smooth muscle cells, endothelial cells, plasma cells, mast cells, plasmacytoid dendritic cells, or 10 other cell types (**Figure 3B**). Bubble plots (**Figure 3C**) demonstrate the differential expression of marker genes in different cell types. The histograms depicting the cell proportions for each sample (**Figure 3D**) and for the overall group (**Figure 3E**) are presented as follows.

### Analyzation of the differential expression of SSR4 in tumor cells and SSR4 immune infiltration

**Figure 4:**
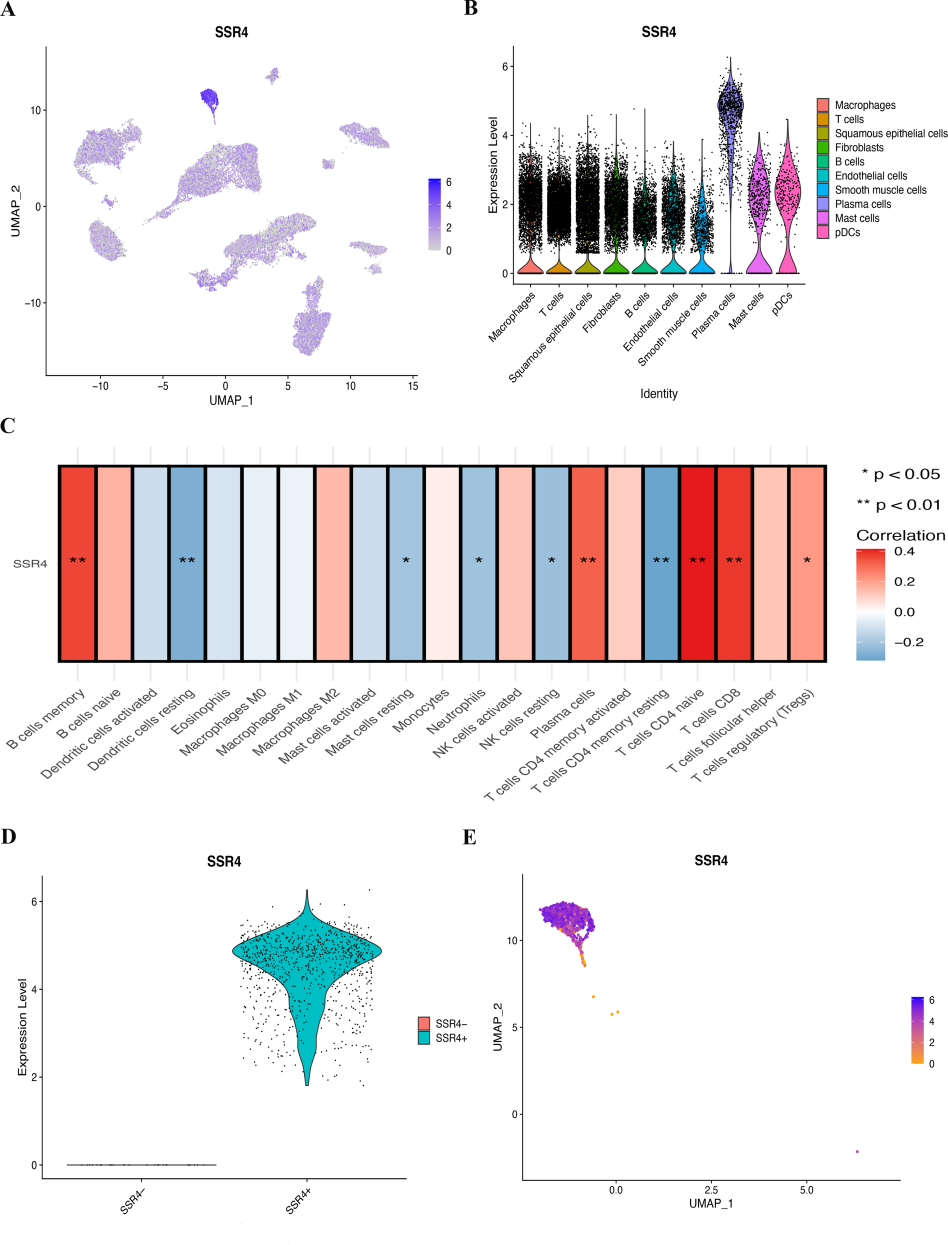
Assessing the possible implication of signal sequence receptor subunit delta (SSR4) at a single-cell level and immune infiltration. **(A, B)** Different cell types’ distribution and expression of SSR4. **(C)** Correlation between SSR4 and immune infiltrating cells based on ssGSEA algorithm. **(D, E)** Two groups of plasma cells were determined based on their expression of SSR4: SSR4+ and SSR4-. (*P < 0.05, **P < 0.01.).

Notably, the highest SSR4 expression was recognized in tumor plasma cells. Consequently, we concentrated our in-depth analysis on tumor plasma cells (**Figures 4A, B**). To investigate the probable implication of SSR4 in tumor plasma tissues, we isolated tumor plasma cell populations and analyzed the interplay between SSR4 expression and biological functions. Using ssGSEA, correlation analysis indicated that many immune cell pairs were either positively or negatively correlated (**Figure 4C**). The plasma cells, CD4 naive T cells, CD8 T cells, B memory cells, and resting dendritic cells exhibited a significant positive correlation (*P*<0.01), while the dendritic resting cells and CD4 memory resting T cells showed a negative correlation.

### SSR4-plasma-DEGs

**Figures 4D, E** depict the categorization of the plasma cell population into SSR4-positive (SSR4+) and -negative (SSR4-) subgroups. Using the FindMarkers function, we identified 187 DEGs between the two subgroups (*P* < 0.05), which were termed SSR4-plasma-DEGs.

### SSR4-plasma-DEGs enrichment analysis

**Figure 5:**
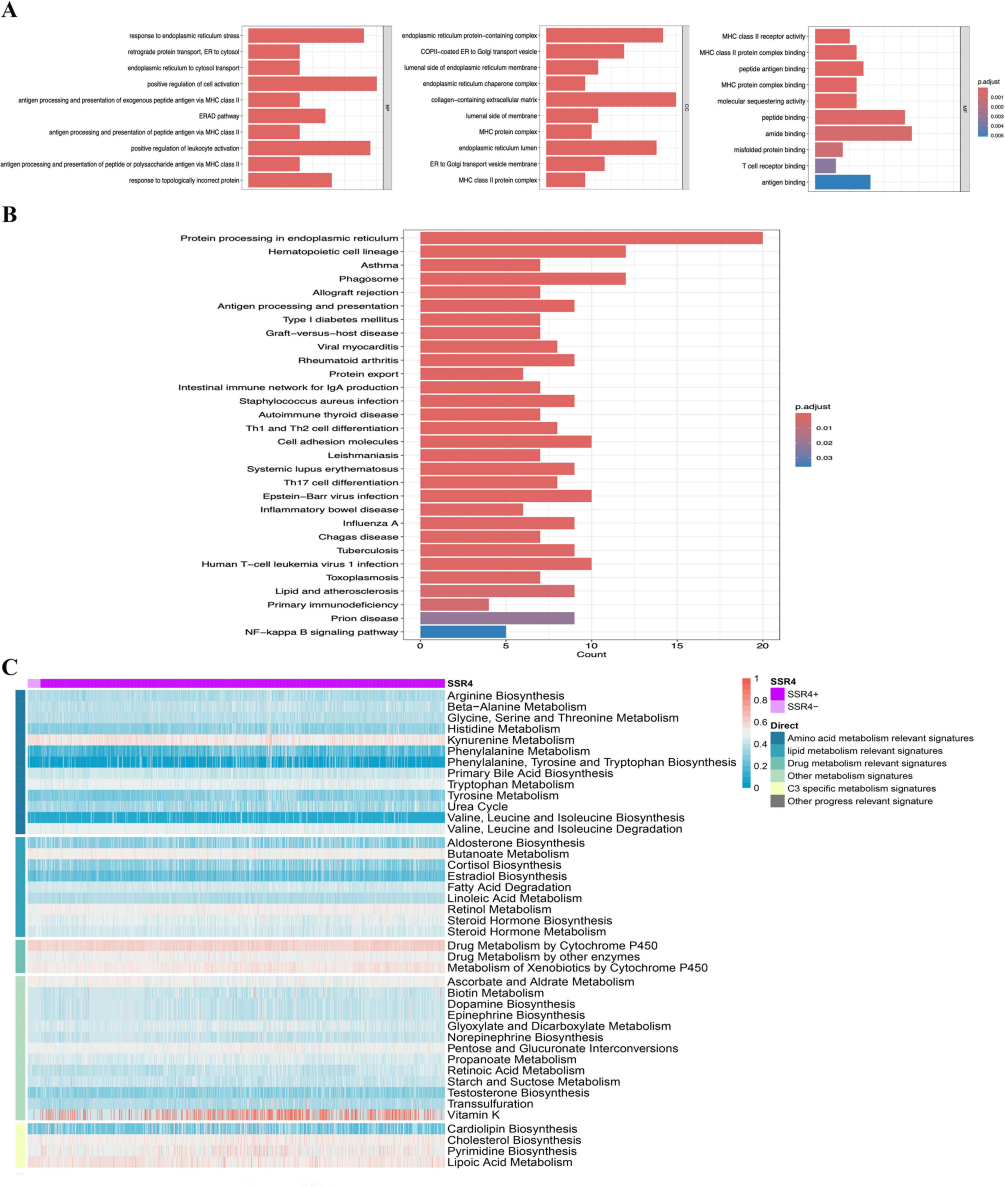
Single-gene functional enrichment analysis. **(A)** Analysis of functional enrichment in GO and **(B)** KEGG: Barplot. **(C)** SSR4+ and SSR4- expressions correlate with metabolic pathways.

Aiming at further investigating the possible biological effects of SSR4-plasma-DEGs, we carried out enrichment analyses on both the overexpressed and suppressed SSR4-plasma-DEGs. GO and KEGG enrichment analyses indicated that SSR4-plasma-DEGs potentially contribute to the positive regulation of cell activation, collagen-containing extracellular matrix, and amide-binding signaling pathways. Additionally, these elements are closely linked to biological functions such as phagosomes, hematopoietic cell lineage, and protein processing in the ER (**Figures 5A, B**). The enriched pathways were statistically significant (*P* < 0.05). The correlation between SSR4 and the metabolic pathways was analyzed at the single-cell level (**Figure 5C**).

### Cell communication analysis of cell interaction patterns in TME of ESCC

**Figure 6:**
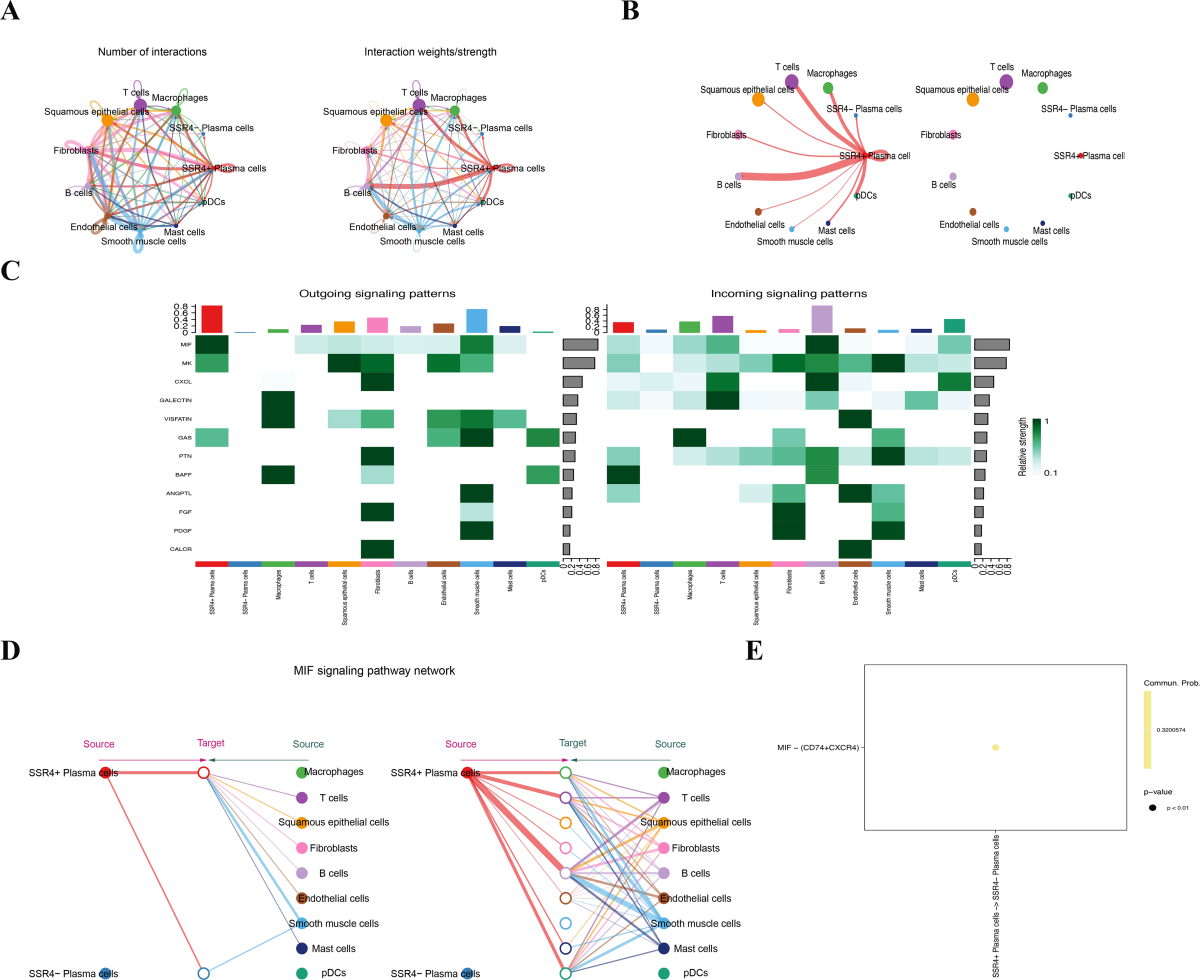
Cell communication suggests that signal sequence receptor subunit delta (SSR4) may affect tumor progression in ESCC via macrophage migration inhibitory factor (MIF) signaling. **(A)** Diagram depicting interaction signal frequency and strength among different cells. **(B)** Plasma-SSR4+ and -SSR4- cells emit different signals. **(C)** A heatmap: Interaction signal intensity between diverse cell types. **(D)** Cells of the Plasma-SSR4+ and -SSR4- lineages interact with each other through the MIF signaling pathway. **(E)** Bubble diagram: Difference between Plasma-SSR4+ and -SSR4- cells.

CellChat analysis revealed different interaction patterns between SSR4+ and SSR4- subsets in ESCC tumor plasma cell populations and other cells in the TME of ESCC. The outcomes manifested differences in the communication signal numbers and intensity between both subgroups as well as other cell types in the TME of ESCC (**Figure 6A**). Thereafter, we analyzed the signal intensity output from the SSR4+ and SSR4- subsets to other cell types in the TME. Although the same two cell subsets exhibited output signals from other cell types, the communication signal intensity between both groups was different (**Figure 6B**). **Figures 6C–E** illustrate a significant difference between the SSR4+ and SSR4- subgroups in the signal pathway output and input patterns like macrophage migration inhibitory factor (MIF). The observed variations in signal strength were especially significant within the MIF pathway, implying that SSR4 may modulate the intricate interaction network between ESCC plasma cells and other cells within the TME by influencing MIF/CD74/CXCR4 signaling. Moreover, expression levels of MIF, CD74, and CXCR4 between ESCC and normal tissues were analyzed with no statistical significance (*P*>0.05) (**Supplementary Figure S4**).

### Construction of a 3D structure model of SSR4 protein and virtual screening of potential drugs

In this study, the 3D structure of the SSR4 protein (SSR4: P51571-nitrosobenzylmethylamine) was predicted. After 50 trials, the binding mechanism of SSR4 was two hydrogen bonds of Leu 63 (binding energy -3.14 kcal/mol, **Supplementary Figure S5**). A docking score of 5.0 kcal/mol indicates potential binding, while a score of 7.0 kcal/mol denotes strong binding (21). This approach aims at predicting the binding modes and affinities between the chemical structures of drugs and proteins.

## Discussion

To our knowledge, we are the first to explore the potential involvement of SSR4 in ESCC. Our findings indicate that gene expression significantly differs between patients with ESCC and healthy controls, suggesting the potential involvement of SSR4 in adverse patient prognosis, as evidenced by data from public databases. Data from RT-qPCR validated this point from four human esophageal cell lines and Tissue microarray slides comprising 110 ESCC and 68 adjacent normal tissue samples were used for further validation using IHC staining. Based on SSR4 expression levels, the study cohort was stratified into low- and high-expression groups comprising 49 and 61 individuals, respectively. The SSR4 expression was related to the N stage, LNM, and AJCC TNM stage. Univariate Cox regression analysis identified patient age, LNM, LMN ratio, N stage, AJCC TNM stage, and SSR4 expression can be considered as potential independent prognostic factors for ESCC. Further multivariate Cox regression analysis confirmed that patient age and LNM ratio were independent prognostic factors for ESCC. Additionally, ESCC patients with low SSR4 expression had a median survival duration that was three months longer than those with high SSR4 expression. On the basis of these results, the possible mechanism of action of SSR4 in ESCC was explored. The data indicated that SSR4 is overexpressed in tumor plasma cells than in other types of cells and it is associated with the regulation of TME, such as immune cells. Upon differentiating between SSR4+ and SSR4- cells, SSR4 may regulate the intricate interaction network between ESCC plasma cells and other cells within the TME, potentially through modulation of the MIF/CD74/CXCR4 pathway.

The SSR4 gene encodes the TRAPδ subunit, and the TRAP complex has four transmembrane subunits (α, β, γ, and δ) in the ER and contributes to protein transport through the ER membrane. The SSR4 protein encoded by this gene is suited in the Xq28 region and organized in a compact head-to-head configuration with the isocitrate dehydrogenase (IDH) 3 (NAD+) gamma gene. A CpG-rich bidirectional promoter regulated both genes, with alternative splicing causing multiple transcript variants (22). Mutations in SSR4 are linked to specific congenital glycosylation disorders, which can manifest as developmental delay, intellectual disabilities, and various physical abnormalities (7, 8, 23). The promoter region of SSR4 is essential for its proper expression, and any alterations may disrupt its function, leading to severe clinical manifestations. IDH genes, which includes IDH1, IDH2, and IDH3, play a pivotal role in cellular metabolism, particularly within the tricarboxylic acid cycle. Mutations in IDH genes, notably IDH1 and IDH2, have been extensively investigated in various cancers such as colon cancer (24), and these mutations may lead to alterations in cellular metabolism and epigenetic regulation, thereby contributing to tumorigenesis. Zhang et al. highlighted the pivotal role of IDH3 in ESCC, specifically, the overexpression of IDH3β (an important regulator of the cell cycle), could enhance glucose uptake, promote cell proliferation, and high expression of IDH3β correlated with poor OS in ESCC patients, suggesting a potential application of IDH3β in prognosis (25). Comprehending the promoter dynamics of IDH genes is essential for the development of targeted therapies, as these genes serve as potential markers and targets for cancer treatment (26). Therefore, disruptions in these promoter configurations can result in severe metabolic and oncogenic consequences, the relationship between the SSR4 gene and the IDH genes in ESCC needs to be further explored, providing novel insights of potential therapeutic targets.

At present, only a few studies on SSR4 expression in tumors have been published. He et al. suggested the potential role of SSR4 in both colon and gastric cancers by regulating immune-infiltrating cells (9). However, although SSR4 was highly expressed in colon tumors, no significant prognostic significance was observed in this study. Another study reported by Amarakoon et al. demonstrated the role of SSR4 by knocking down human colorectal cancer cell lines (HCT 116, SW480), revealing a marked decrease in viability, and increased arrests in the S and G1 phases (27). The SSR4 gene was involved in a stable prognostic model containing eight genes based on ER stress in lung adenocarcinoma, as reported by Yang et al. (28). In our study, despite confirming the high expression of SSR4 by RT-qPCR and IHC staining (6), SSR4 was related to LNM and poor prognosis. It is the first time to report the role of the SSR4 gene in ESCC patients. Furthermore, we further explored the SSR4 gene and its correlation with several immune cells, such as plasma cells, CD4 naive T cells, and CD8 T cells. Currently, there is no literature elaborating on how the SSR4 gene directly regulates the cancer microenvironment in ESCC. Therefore, we speculate that this gene may be a biomarker and therapeutic target that may affect the TME of ESCC.

Focusing on the SSR4+ and SSR4- cell subtypes, SSR4 expression levels were closely related to the interaction patterns that existed between tumor plasma cells as well as other cell subtypes, especially in the MIF/CD74/CXCR4 pathway. The MIF/CD74 axis is a pathway that regulates B lymphocyte chemotaxis (29). The MIF functions as a key upstream regulator of the innate immune response besides protecting against apoptosis induced by oxidative stress (30). The CXCR4 overexpression participates in tumor growth, metastatic dissemination, and cancer relapse (31). The interaction that existed between MIF, its receptor CD74, and the chemokine receptor, CXCR4, is crucial in tumor biology, particularly cancer progression and metastasis. The MIF/CD74 axis is pivotal for promoting cancer cell survival and proliferation, whereas CXCR4 is involved in mediating the metastatic spread of tumors through its ligand stromal cell-derived factor-1 (32, 33). In breast cancer, colorectal cancer, and rhabdomyosarcoma, the MIF/CD74/CXCR4 axis influences tumor cell behavior and supportive stromal cell recruitment (34–36). In addition, MIF plays a key role in disturbing lipid metabolism in laryngeal carcinoma (37). However, studies on the implication of the MIF/CD74 axis in ESCC are scarce. In this research, no statistical differences in the expression levels of MIF, CD74, and CXCR4 were found between tumor and normal groups. Therefore, we speculate that SSR4 may facilitate ESCC progression via the MIF/CD74 axis. Given that our identified genes were selected through an ESCC prognostic model constructed based on glucose metabolism-related gene sets, and considering that altered glucose metabolism is a contributing factor to ESCC progression. While we have found a significant correlation between SSR4 and the tumor immune microenvironment, the direct involvement of SSR4 in regulating glucose metabolism within this context remains to be elucidated.

Our study had some limitations. First, in the process of exploring the potential role of SSR4 in ESCC, our research data were obtained from public databases, and our findings necessitate additional *in vivo* and *in vitro* verification. Specifically, since the SSR4 gene has been explored in tumor plasma cells, the interaction between plasma cells and squamous epithelial cells requires further exploration, such as co-culturing plasma cells with squamous epithelial cells and knocking down the key gene. Second, although the cellular composition of tumor tissues and normal tissues was presented, a quantitative analysis, such as the Ro/e diagram, may provide a more effective representation of the issue. Moreover, our study relied solely on scRNA-seq as well as bulk RNA data, other scRNA-seq data of ESCC such as GSE196756 issued in 2023, along with additional studies, especially multiomics, spatial transcriptomics, and spatial metabolomics analyses, which are needed to study how SSR4 therapeutically affects ESCC. Another limitation of this study is the absence of an internal control gene, such as PD-1/PD-L1, which is known to be differentially expressed in ESCC. Due to the retrospective nature of this study and the limited availability of tissue samples, we were unable to incorporate PD-1/PD-L1 as an internal control. The inclusion of such a control could have further standardized our RNA-seq and IHC data, reducing potential technical variability and enhancing the interpretability of our results. Consequently, the findings need to be approached with caution.

## Conclusion

In conclusion, SSR4 was significantly overexpressed in ESCC and was closely connected to patients’ OS. The SSR4 expression may correlate with the N stage, LNM, and AJCC TNM classification stage. The SSR4 gene may play a critical role in the regulation of the tumor microenvironment of ESCC patients. Differentiation between SSR4+ and SSR4- cells suggests that SSR4 might regulate the interactions between ESCC tumor plasma cells and TME, possibly by modulating the MIF/CD74/CXCR4 axis.

## Data Availability

The datasets presented in this study can be found in online repositories. The names of the repository/repositories and accession number(s) can be found below: https://www.ncbi.nlm.nih.gov/geo/, GSE188900.

## Supplementary material

Supplementary Figure 1 Expression of PD-1/PD-L1 in esophageal squamous cell carcinoma (ESCC) patients and the control group. (*** *P* < 0.001, ns, no statistical significance.).

Supplementary Figure 2 Kaplan-Meier curves of the overall survival based on the signal sequence receptor subunit delta (SSR4) expression.

Supplementary Figure 3 Quality control and normalization of the ESCC scRNA-seq data. **(A-B)** Number of unique molecular identifiers/cell (nCount_RNA), gene count/cell (nFeature_RNA), mitochondrial gene count/cell (percent.mt), and ribosomal gene count/cell (percent.ribo). **(C)** Gene scatter plots showed 2000 genes with high variability in red, and the top 10 genes were flagged. **(D)** Harmony reduces batch effect by iteratively clustering similar cells within each cluster while maintaining batch diversity.

Supplementary Figure 4 Expression levels of the MIF gene, CD74 gene, and CXCR4 gene between the tumor and normal tissues.

Supplementary Figure 5 Molecular docking of signal sequence receptor subunit delta (SSR4).
