# Supplementary figures and images for: Unveiling SSR4: a promising biomarker in esophageal squamous cell carcinoma
Source: Front Immunol. 2025 Feb 24;16:1544154. doi: 10.3389/fimmu.2025.1544154 (PMC11891195; doi:10.3389/fimmu.2025.1544154)

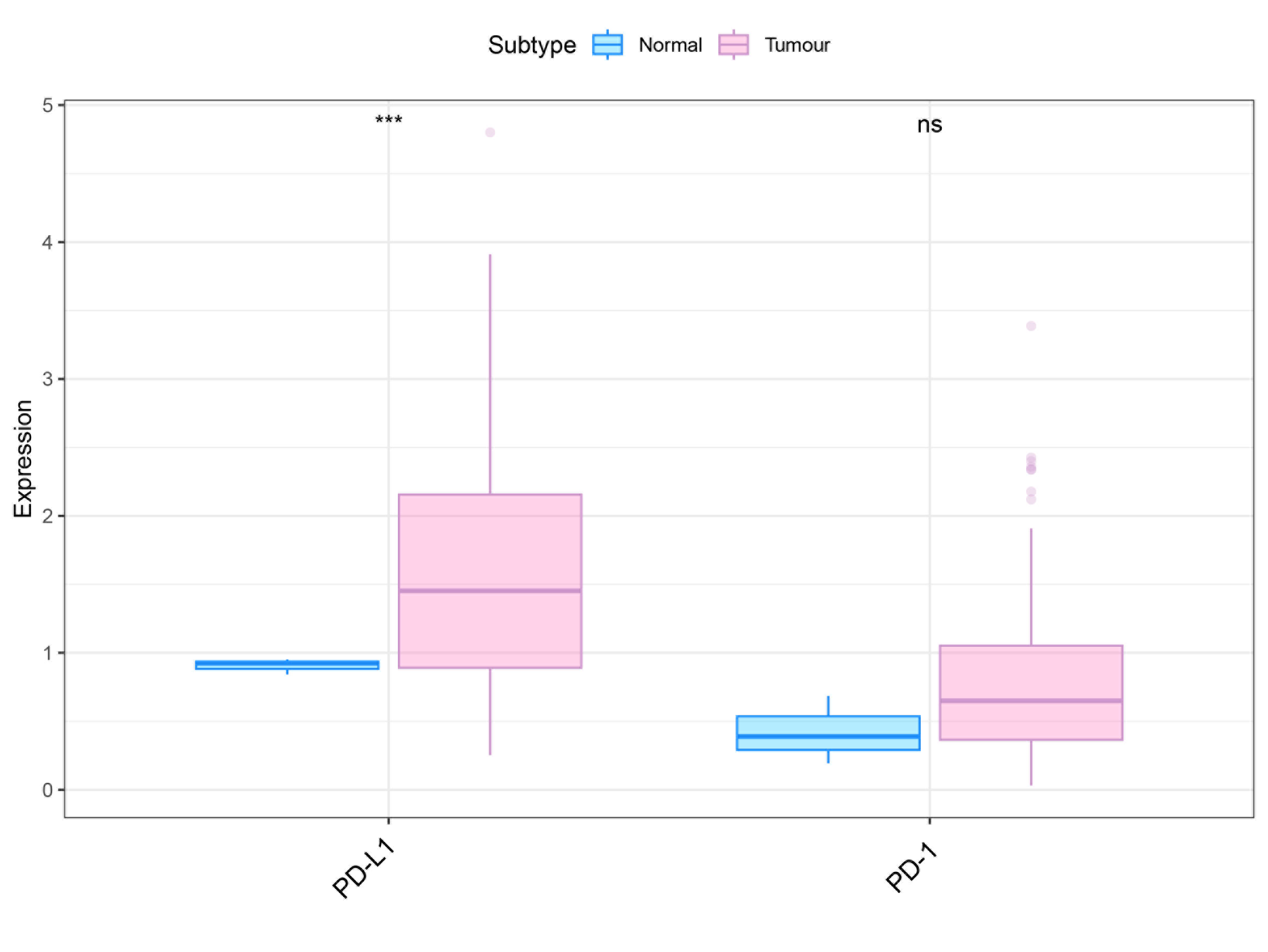

Supplement: Supplementary Figure 1 — Expression of PD-1/PD-L1 in esophageal squamous cell carcinoma (ESCC) patients and the control group. (*** P < 0.001, ns, no statistical significance.). [file Image1.tif]

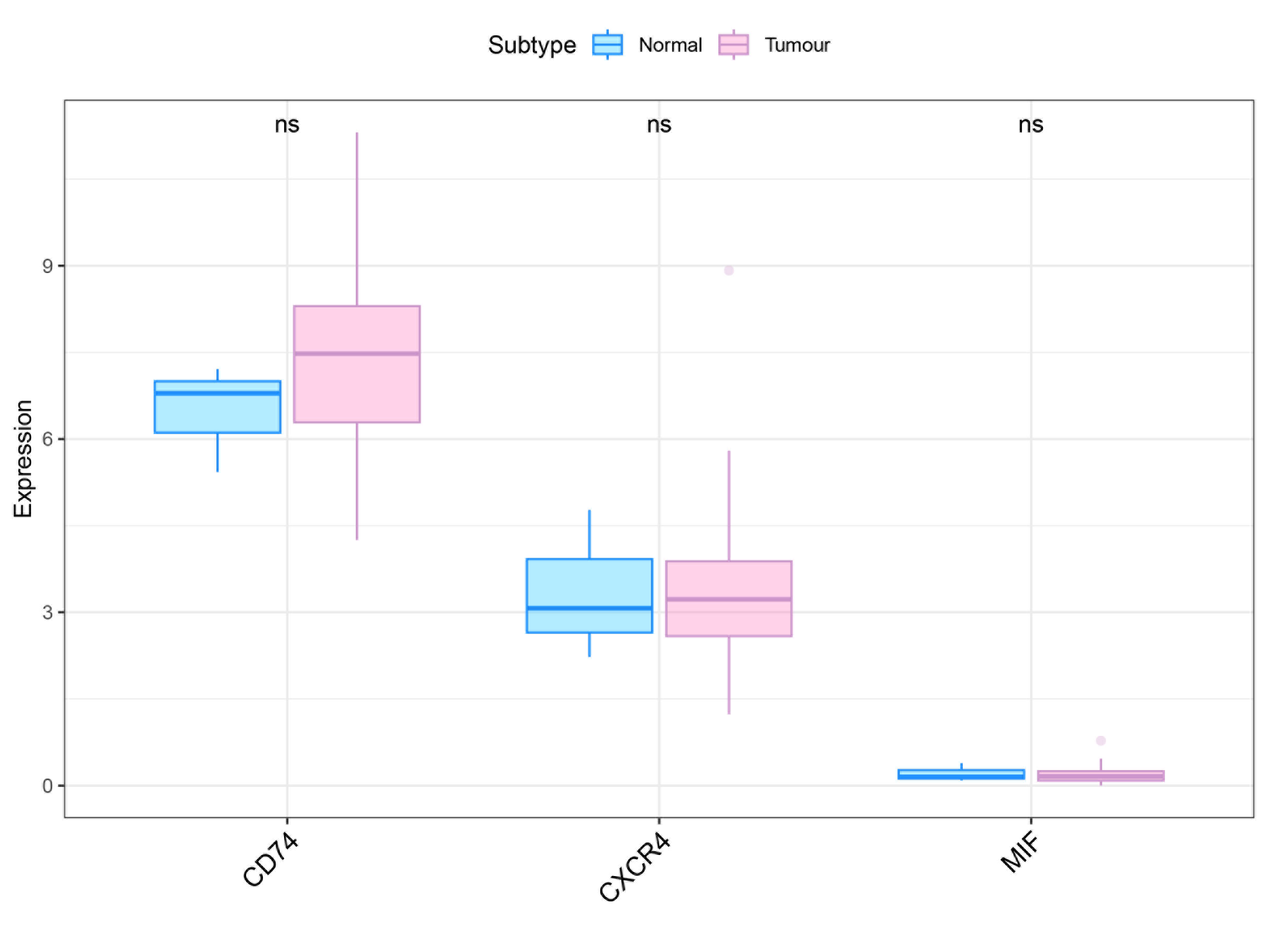

Supplement: Supplementary Figure 4 — Expression levels of the MIF gene, CD74 gene, and CXCR4 gene between the tumor and normal tissues. [file Image4.tif]
