# Supplementary material for: Unveiling SSR4: a promising biomarker in esophageal squamous cell carcinoma
Source: Front Immunol. 2025 Feb 24;16:1544154. doi: 10.3389/fimmu.2025.1544154 (PMC11891195; doi:10.3389/fimmu.2025.1544154)
Supplement: Supplementary file 6 [file Table1.docx]

**Table S1.** Primers of RT-qPCR

| Gene | Forward (5‘-3’) | Reverse (5‘-3’) |
| --- | --- | --- |
| β-actin | TGGCACCCAGCACAATGAA | CTAAGTCATAGTCCGCCTAGAAGCA |
| SSR4 | TCACCCCTTCCTACTACACCA | GACCCTGTTCTTGCATGTCAG |
